# The Role of Stem Cells as Therapeutics for Ischaemic Stroke

**DOI:** 10.3390/cells13020112

**Published:** 2024-01-06

**Authors:** Jingyuan Ya, Jessica Pellumbaj, Arshad Hashmat, Ulvi Bayraktutan

**Affiliations:** Academic Unit of Mental Health and Clinical Neurosciences, Queens Medical Centre, School of Medicine, University of Nottingham, Nottingham NG7 2UH, UK

**Keywords:** stem cells, cell-based therapy, endothelial progenitor cells, ischaemic stroke

## Abstract

Stroke remains one of the leading causes of death and disability worldwide. Current reperfusion treatments for ischaemic stroke are limited due to their narrow therapeutic window in rescuing ischaemic penumbra. Stem cell therapy offers a promising alternative. As a regenerative medicine, stem cells offer a wider range of treatment strategies, including long-term intervention for chronic patients, through the reparation and replacement of injured cells via mechanisms of differentiation and proliferation. The purpose of this review is to evaluate the therapeutic role of stem cells for ischaemic stroke. This paper discusses the pathology during acute, subacute, and chronic phases of cerebral ischaemic injury, highlights the mechanisms involved in mesenchymal, endothelial, haematopoietic, and neural stem cell-mediated cerebrovascular regeneration, and evaluates the pre-clinical and clinical data concerning the safety and efficacy of stem cell-based treatments. The treatment of stroke patients with different types of stem cells appears to be safe and efficacious even at relatively higher concentrations irrespective of the route and timing of administration. The priming or pre-conditioning of cells prior to administration appears to help augment their therapeutic impact. However, larger patient cohorts and later-phase trials are required to consolidate these findings.

## 1. Introduction

Stroke continues to be one of the leading causes of mortality and morbidity in the world, with around thirty-two thousand stroke-related deaths in England alone each year [1]. As the population ages, the prevalence of stroke-related death and disability will continue to rise, presenting a substantial public health burden. Stroke occurs when cerebral blood supply is disrupted as a result of an occlusion (ischaemic strokes) or rupture (haemorrhagic strokes) of an artery leading to, within, or on the surface of the brain. The brain is particularly susceptible to damage when it is starved of oxygen and glucose even for shorter periods of time, leading to the sudden appearance of contralateral hemiparesis, speech difficulties, confusion, visual disturbances, balance problems, and a severe headache. In chronic phases, the patients manifest persistent aphasia, amnesia, and problems with emotional functioning. This marked reduction in the quality of life is a catalyst for the stroke research community to discover new agents or interventions for stroke.

While a range of effective prophylactic medicines exist, including aspirin, clopidogrel, warfarin, and other anticoagulants, the current curative therapeutic options are restricted to thrombolysis, thrombectomy, or bridging treatment. Thrombolysis is realised by intravenous (IV) administration of recombinant tissue plasminogen activator (rt-PA) to eligible patients to restore cerebral blood flow. Though proven to be safe and effective in improving clinical outcomes at three months [2], the therapeutic window for thrombolysis is limited. To minimise the damage to ischaemic penumbra, rt-PA must be administered within the first 4.5 h of an ischaemic stroke [3]. Beyond this therapeutic window, intravenous thrombolysis (IVT) may further compromise the integrity of the blood–brain barrier (BBB), consequently giving rise to symptomatic intracerebral haemorrhage [4]. Endovascular thrombectomy (EVT) is an invasive procedure which involves the insertion of a catheter into an artery to surgically remove thrombus for recanalisation. Beneficial effects of EVT were determined in patients with acute ischaemic stroke who received treatment 6 to 24 h after they had last been known to be well [5]. In addition to the narrow time window, patients with most types of active haemorrhage are not eligible for both IVT, EVT, or other anticoagulatory treatments [6].

In this regard, stem cells, with their self-renewing capabilities and capacity to differentiate and repair damaged tissue, present an exciting alternative. Instead of targeting the cause of stroke, stem cells act to reverse or remedy the pathological damage caused by ischaemic damage. By presenting a therapeutic option beyond the acute phase of stroke, stem cells may be of significant value to extend the interventional strategies to all patient profiles. This review examines a selection of different stem cell types and their therapeutic relevance in ischaemic stroke. The niches discussed in this paper include mesenchymal stem cells (MSCs), endothelial progenitor cells (EPCs), haematopoietic stem cells (HSCs), and neural stem cells (NSCs). The majority of the clinical studies cited in this paper were performed with patients with moderate to severe ischaemic stroke affecting the middle cerebral artery or carotid artery. In the overwhelming majority of the pre-clinical studies, a transient or rarely a permanent middle cerebral artery occlusion (MCAO) model of ischaemic stroke was employed. 

## 2. Pathology of Ischaemic Stroke

It is essential to comprehend different mechanisms and networks involved in the pathogenesis and outcome of stroke to appreciate the therapeutic use and value of stem cells. The mechanisms discussed below occur in many different cell types ranging from nervous tissue, involved in sensory and motor communications, to cells of the BBB, responsible for protecting the CNS.

### 2.1. Excitotoxic Cell Death

The hypoxia that occurs in the immediate aftermath of an ischaemic attack triggers excitotoxic cell death. Hypoxic conditions downregulate ATP production by inhibiting plasma membrane Na^+^/K^+^/ATPase and Ca^2+^/ATPase pumps [7,8]. Receptor malfunction increases intracellular Na^+^ and Ca^2+^, causing cellular depolarisation and the propagation of action potentials. Na+ influx results in K^+^ efflux, further stimulating peri-infarct depolarisation. High levels of intracellular Ca^2+^ trigger glutamate exocytosis into the synaptic cleft; this accretion stimulates postsynaptic glutamate receptors, further increasing intracellular Ca^2+^ in the postsynaptic neurone [9,10]. An excessive Ca^2+^ load results in mitochondrial dysfunction, stimulating proteolysis and NADPH oxidase enzyme induction, triggering oxidative stress accompanied by the excessive release of reactive oxygen species (ROS). Once generated, ROS promote inflammatory mechanisms by attracting cytokines and leukocytes to infiltrate the brain as the BBB degrades [11,12,13,14]. Microglial cells, which are activated under oxidative stress, along with cytokines, also recruit matrix metalloproteinases (MMPs), a family of protease enzymes, further aiding local inflammation of ischaemic tissue [15]. Both activated microglia and reactive astrocytes are major components of the immune system in the brain, and the crosstalk between them reinforces the release of several proinflammatory factors, including IL-1β, IL-6, TNF-α, IL-15, and MMPs [16,17]. This homeostatic upset, inflammation, and uncontrolled enzymatic degradation inevitably damages the cellular structure and function and adversely affects the surrounding microenvironment. 

### 2.2. Apoptosis, Necrosis, and Necroptosis Pathways 

A lack of Ca^2+^ homeostasis also stimulates numerous cellular death pathways. Ischaemia induces apoptosis via the release of cytochrome C from dysfunctional mitochondria followed by the activation of caspase-3 and the downstream hydrolases [18]. The cell enters the execution phase of apoptosis; the cytoplasm begins to shrink and display cytomorphological changes, including nuclear condensation [19]. Alternatively, the cell may undergo necrosis. This is often described as premature cell death and occurs due to Na^+^ influx accompanying Na^+^/K^+^ pump and Ca^2+^/ATP pump failure. Intracellular Na^+^ and Ca^2+^ aggregation leads to cellular oedema, swelling, and loss of lysosomal membrane integrity and cell rupture. Exposed cellular components attract digestive molecules for cell lysis, further contributing to local inflammation. It is noteworthy that unlike apoptosis, necrosis is independent of caspase activity [20,21]. Another recognised route of cell death in stroke is necroptosis, otherwise known as ‘programmed necrosis’. Through currently unidentified mechanisms, necroptosis appears to be regulated by receptor-interacting protein kinase 1 (RIPK1) and involves the swelling of the cell and lysosomal rupture (mimicking necrosis) and similar to apoptosis requires caspase and cytochrome C activity [22]. 

Autophagy or auto-phagocytosis is another degradation pathway often witnessed in ischaemic stroke. Autophagy is the breakdown and phagocytosis of cellular organelles to maintain homeostasis [15,23]. Its catabolic effects allow for the emergency production of energy and nutrients during hypoxia and other stressful conditions. Though autophagy is generally recognised as a protective pathway, evidence linking it to neurodegeneration also exists [24]. 

## 3. Blood–Brain Barrier 

The BBB, an integral component of the neurovascular unit, regulates the selective passage of compounds between the blood and the brain parenchyma [15,25]. The BBB consists of pericytes, astrocytes, and endothelial cells (ECs) and is paracellularly sealed by tight junctions (TJs). These protein complexes are primarily composed of the transmembrane proteins claudins, occludins, junction adhesion molecules (JAMs), and zone occludens (ZO), an accessory protein responsible for manoeuvring cytoskeletal interactions [26]. The claudin family demonstrate a variety of transmembrane domains, of which the claudin-5 isoform is most greatly expressed, showing direct responsibility in tightening the BBB against small molecules (<800 Da) [26,27]. Occludins form dimers and oligomers, aiding paracellular permeability and stabilising barrier function, to which JAMs provide further support. The degradation of these tight junction constituents, catalysed by activated MMPs, compromises the BBB. Hypoxia and exaggerated local cytokine availability augment MMP expression, with elevated MMP-2 and MMP-9 levels identified in stroke patients [15,28,29,30,31,32]. The restoration of BBB integrity during the post-ischaemic period by MMP inhibition highlights this relationship [33,34]. Furthermore, the autophagy of claudin-5 is associated with significant increases in the solute permeability of the BBB [15,30]. The decreased expression of ZO-1, occludin, and claudin-5 in senescent ECs leads to the impairment of BBB integrity [35]. The disruption of other TJ factors, such as the JAM VE-cadherin, results in BBB compromise [27]. Furthermore, the pathology of pericytes which mature and maintain the BBB and astrocytes, involved in the maintenance of osmotic BBB conditions, further precipitate BBB breakdown [36]. 

The destruction of the basement membrane is another pathology to consider in ischaemic stroke. The basement membrane is a non-cellular complex consisting of a sheath of extracellular matrix and a series of proteins, namely collagen IV, nidogen, perlecan, agrin, and laminin. Although how the basement membrane becomes damaged during ischaemic stroke remains vague, it is presumed that the membrane undergoes dissolution, thereby exacerbating the loss of BBB and vascular integrity [37,38,39].

## 4. Stem Cell as Therapeutics

**Figure 1:**
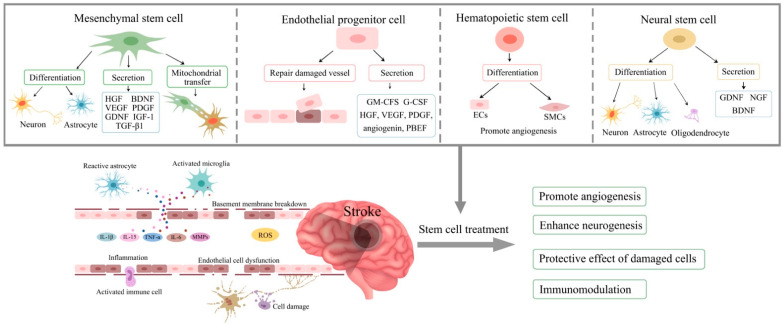
Mechanisms of stem cell therapy for ischaemic stroke. Excitotoxic cell damages, activation of immune cells, inflammatory reaction, breakdown of blood–brain barrier, mitochondrial dysfunction, and oxidative stress are involved in the pathophysiology of stroke. Stem cells have the potential to ameliorate these processes via differentiation into various cells to replace the damaged cells and secrete cytokines and growth factors to promote angiogenesis, neurogenesis, and immunomodulation. HGF, hepatocyte growth factor; BDNF, brain-derived neurotrophic factor; VEGF, vascular endothelial growth factor; PDGF, platelet-derived growth factor; GDNF, glial cell-derived neurotrophic factor; IGF-1, insulin-like growth factor 1; TGF-β1, transforming growth factor beta-1; GM-CFS, granulocyte-macrophage colony-stimulating factor; G-CSF, granulocyte colony-stimulating factor; PBEF, pre-B cell-enhancing factor; ECs, endothelial cells; SMCs, smooth muscle cells; NGF, nerve growth factor; IL, interleukin; TNF-α, tumour necrosis factor alpha; MMPs, matrix metalloproteinases; ROS, reactive oxygen species.

A literature search using the key MeSH terms “stem cells”, “ischaemic stroke”, “stroke pathology”, “mesenchymal stem cells” (MSCs), “endothelial progenitor cells” (EPCs), “haematopoietic stem cells” (HSCs), and “neural stem cells” (NSCs) on the PubMed database identified relevant studies. Nottingham University search and Google Scholar were also used to collect pertinent studies. This paper has critically evaluated a variety of pre-clinical and clinical studies to ascertain the role of stem cells as therapeutics for stroke. The mechanisms of stem cell therapy in ischaemic stroke is summarised in Figure 1. 

## 5. Mesenchymal Stem Cells

MSCs are multipotent adult stem cells with the ability to differentiate into various cell types within the mesodermal lineage, including bone cells, cartilage, muscle cells, and skin cells. Despite their limited capacity to differentiate, evidence exists regarding trans-differentiation along the ectodermal lineage into neural cells and along the endodermal lineage into hepatocytes [40]. MSCs are isolated from bone marrow (BM), adipose tissue, Wharton’s Jelly (WJ) in umbilical tissue, amniotic fluid, and dental pulp [41]. Since the differentiation capacity decreases with age, WJ-derived MSCs show greater potential to differentiate than MSCs derived from other sources. Even so, BM-derived MSCs and adipose tissue-derived cells are most utilised in stem cell therapy due to their plasticity, availability, and immunomodulatory properties compared to other sources. Their lack of major histocompatibility complex (MHC)-I and MHC-II antigens allows for allogenic administration without the risk of transplant-induced teratoma formations [42,43].

The paracrine signalling of the MSC secretome induces behavioural, mechanical, and chemical changes in adjacent cells. These changes result in angiogenic, neovascular, and anti-inflammatory effects. The secretome of MSCs also contains factors responsible for directing the fate of other stem cells [44,45,46,47]. The secretome of MSCs includes growth factors, cytokines, chemokines, and various anti-inflammatory agents, including vascular endothelial growth factor (VEGF), brain-derived neurotrophic factor (BDNF), basic fibroblast growth factor (bFGF), hepatocyte growth factor (HGF), insulin-like growth factor (IGF-1), platelet-derived growth factor (PDGF), transforming growth factor beta-1 (TGF-β1), osteopontin (OPN), and interleukin-6 (IL-6) [41,48,49]. Extracellular vesicles play an important role in transferring these molecules between neighbour cells, allowing for the co-ordination of regenerative mechanisms and cellular migration, proliferation, and homing to the site of injury [50]. 

The homing of MSCs, and stem cells in general, to the site of injury is an important step in tissue regeneration. The increased availability of OPN after tissue injury is one of the key factors that regulates both MSC homing and migration. OPN mitigates stress-imposed alterations in cellular morphology by suppressing actin stress fibre formation which in turn allows dynamic movement and relocation in which integrin β-1, FAK, and ERK pathways appear to play a role [51]. The activation of the stromal-derived factor-1 (SDF-1)/CXC chemokine receptor-4 (CXCR4) pathway, on the other hand, has been implicated in suppression of MSC migration [49]. 

There is evidence that the anti-inflammatory activities of MSCs are mediated through mechanisms involving VEGF signalling and concomitant reductions in the expression of tumour necrosis factor-α (TNF-α) and transcription factor NF-κB [52]. Indeed, TNF-α modulates the composition of the MSC secretome [12,50,53], which may influence the endothelial cell migration, differentiation, and proliferation and affect the extent of angiogenesis. It is assumed that bFGF, VEGF, TGF-β, HGF, and IL-6 signalling are closely involved in these paracrine effects [54]. In support of this, the transplantation of adipose-derived MSCs to MCAO rats has been shown to promote angiogenesis and encourage behavioural recovery and transplantation of BM-MSCs to cerebral infarcts has been shown to increase VEGF levels, resulting in ERK phosphorylation and the repair of white matter damage to help cognitive recovery [55,56]. Furthermore, the expression of VEGF and IGF-1 in ischaemic tissue supports the regeneration of astrocytes, microglia, and oligodendrocytes [57].

BM-MSCs can also stimulate the release of BDNF in local parenchyma to activate the Akt/PI3K pathway which meditates cellular growth, proliferation, and angiogenesis [58]. BDNF’s role in neuroregeneration is further recognised in astrocytic Akt/mTOR signalling in the recruitment of additional astrocytes for nerve injury repair [59]. The co-administration of BM-MSCs with regulators of stem cell differentiation and migration, butylidenephthalide and sodium ferulate, appears to enhance the expression of astrocyte-derived VEGF and BDNF in vivo, further supporting the role of MSCs in promoting angiogenesis [59]. Furthermore, by attenuating the degree of stroke-induced calcineurin (CaN) hyperactivation, MSCs rescue neurones undergoing apoptosis and improve neuronal activity in rodents [60]. Similarly, the co-application of MSCs with erythropoietin (EPO) triggered neurogenesis and cellular proliferation along the lateral ventricles in rats subjected to transient MCAO [61]. It is of note that studies conducted with animal models of other related diseases like myocardial infarction also provide evidence for the regenerative properties of MSCs [62,63]. 

MSC-induced neuroplasticity has also been observed in clinical settings where the injection of BM-MSCs through the IV route led to an increase in the number of cluster activations in Brodmann areas BA4 and BA6 and improved the clinical outcome, as evidenced by the Barthel Index (BI) and Fugl-Meyer (FM) scores [64]. A randomised clinical trial also pinpoints the neuroplastic effects of MSCs in recovering motor function in ischaemic stroke patients [65]. The study was consistent with pre-clinical and clinical trials documenting that the IV administration of autologous BM-MSCs is safe and feasible without the precipitation of tumours or related adverse events (AEs). The trial provided further evidence for the paracrine action of MSCs associated with improvements in behavioural and motor abilities and an increase in task-related primary motor cortex (MI) activities. Furthermore, observations of behavioural and physiological improvements two years after treatment, the final point of assessment in the study, demonstrate a long-term benefit in these patients and propose MSC therapy as a chronic management strategy for ischaemic stroke [65]. 

Accumulating recent evidence indicates that the transplantation of MSCs through different routes is safe and efficacious in improving patients’ functional outcome [66,67]. Indeed, while the intracerebral administration of BM-MSCs attenuated disease severity and improved the outcome, as evidenced by changes in the National Institutes of Health Stroke Scale (NIHSS) and fine motor scores [68], the intra-arterial infusion of MSCs during the subacute phase of the disease was coupled with a better clinical outcome, defined by a modified Rankin Scale (mRS) score < 2 in the treatment arm versus the control group [69]. Patient improvements have also been witnessed in clinical settings assessing the efficacy of NSC and MSC co-transplantation, where treatment conferred the refinement of speech, balance, and muscular control [70,71]. Table 1 summarises the details of the clinical trials, indicating the safety of treatments with MSCs. Taken together, these studies suggest that larger trials of bigger sample sizes with longer, more extensive follow-ups are necessary in order to comment on treatment efficacy reliably.

Another mechanism involved in the regenerative role of MSCs is rather unique in that MSCs can rescue cells injured due to mitochondrial dysfunction via mitochondrial transfer. The presence of tunnelling nanotubes transferring mitochondria from MSCs to damaged H9c2 cardiomyocytes to restore mitochondrial function has been shown in an in vitro ischaemia/reperfusion model with fluorescent microscopy [72]. The metabolic benefits relating to MSC mitochondrial transfer have also been reported in in vivo and in vitro settings in response to oxidative stress [73]. Similar results also show growing evidence favouring MSC use in the treatment of stroke [74]. 

To abet their regenerative properties, MSCs can be primed or pre-conditioned, which involves preparing cells for a specific purpose, including lineage-specific differentiation, through either epigenetic and morphological modifications or the manipulation of the cell culture environment [75]. Studies comparing MSC efficacy before and after priming attribute an important role to cell priming in achieving higher therapeutic efficacy [75,76]. 

## 6. Endothelial Progenitor Cells (EPCs)

EPCs are circulating stem cells of endothelial origin. They migrate and accumulate in areas of vascular injury to help repair damaged vasculature through both neovascularisation and vascular remodelling. Due to their ability to detect and replace the damaged cerebral endothelial cells and restore BBB integrity by differentiating into mature endothelial cells, they are regarded as an important therapeutic for the management of ischaemic stroke [77,78]. An insufficient number and dysfunction of EPCs impairs vascular homeostasis and accelerates vascular disease [79,80]. EPCs are released into circulation by bone marrow in response to an ischaemic injury. They are isolated from the mononuclear cell (MNC) population through the use of specific antigens targeting endothelial cell maturity (e.g., KDR+), immaturity (e.g., CD133+), and stemness (e.g., CD34+) amongst a non-haematopoietic cell (CD45-) population [81]. To obtain cells that can be used for therapeutic purposes, MNCs are cultivated using specific endothelial cell media supplemented with a range of factors, including fibroblast growth factor (FGF), VEGF, insulin-like growth factor (IGF), hydrocortisone, ascorbic acid, and heparin [82,83,84]. The exogenous addition of EPCs repairs the integrity of an in vitro BBB model under OGD conditions and attenuates ischaemia-evoked oxidative stress and the apoptosis of endothelial cells [77,85,86]. Besides administering the stem cells directly, the condition media of outgrowth endothelial cells were found to negate the deleterious effects of TNF-α on BBB function in an in vitro tri-culture model of the BBB [87]. EPCs are able to secret a wide range of substances to regulate angiogenesis, migration, proliferation, and antipoptosis, including angiogenin, HGF, PDGF, VEGF, and PBEF [88]. A clinical study indicated that post-stroke patients have elevated levels of TNF-α, and an in vitro study determined that TNF-α significantly impairs the function and integrity of the BBB [87,89]. Aside from their therapeutic potential, EPCs can also be used as diagnostic and prognostic biomarkers. Significantly higher numbers of circulation EPCs were found in patients in the acute and subacute phase after ischaemic stroke, compared to healthy volunteers, indicating that the EPC number can be used to monitor the recovery/progression of ischaemic stroke. However, no correlation was determined between EPC counts and patients’ outcome [90]. However, recent observation of a close correlation between baseline CD34+KDR+ and CD133+KDR+ counts and the outcome of stroke supports the idea that these particular EPC subtypes can be used as potential prognostic markers for ischaemic stroke [91]. Low EPC concentrations have been reported in chronic stroke patients and those with cardiovascular risk factors [92]. 

EPCs in culture produce two distinct types of cells: early EPCs (eEPCs) and outgrowth ECs (OECs) or endothelial colony-forming cells (ECFCs). eEPCs represent an immature (CD133+) population of EPCs, with little proliferative capacity, appearing early in culture (three to four days). In contrast, OECs appear late in culture (two to four weeks) and demonstrate maturity and commitment to differentiation [93]. eEPCs and OECs can also be distinguished by their different morphology in that while eEPCs show a spindle-shaped morphology, OECs manifest the classical endothelial phenotype of cobblestone morphology [81,88]. Moreover, eEPCs express haematopietic markers CD45 and CD14 while OECs express endothelial markers CD31, CD146, and CD105 and stemness marker CD34 [94,95]. 

A variety of agents, including VEGF, NO, EPO, SDF-1, and active MMP-9, regulate the mobilisation of EPCs from the BM into circulation [96,97]. VEGF, a key mediator of angiogenesis, stimulates EC proliferation, migration, and tube formation, eventually giving rise to new blood vessels and capillary networks [98,99,100]. In vivo and in vitro experiments using rat-spleen-derived EPCs have documented that connexin-43 plays a pivotal role in EPC differentiation while VEGF promotes EPC proliferation and vascular repair [100]. VEGF meditation of angiogenesis explains just one of the mechanisms by which EPCs promote neovascularisation [101].

The trafficking of EPCs, co-ordinated by SDF-1, is supported by the results of another in vivo investigation looking at the relationship between hypoxia-inducible factor-1 (HIF-1) and SDF-1 in ischaemic mice [29]. The study concluded that HIF-1 directly regulated *SDF-1* gene expression in ischaemic tissue and that the migration and adhesion of EPCs to sites of injury was supported via CXCR4 and SDF-1 binding. A different study exploring the dynamics of EPC-induced vascular remodelling and angiogenesis provides evidence for IL-6 which activates gp80/130 signalling pathways, including downstream ERK1/2 phosphorylation [102]. Most importantly, this study confirmed that IL-6 stimulates EPC proliferation, movement, and adhesion. The manipulation of ischaemic matrigel models confirm this relationship, where IL-6 influence was credited with significantly promoting EPC capillary-like tube formations [102]. 

Nitric oxide generated in endothelial cells by endothelial nitric oxide synthase (eNOS) is another important molecule that co-ordinates EPC proliferation and migration and inhibits apoptosis and platelet aggregation [103,104]. Observation of an impaired ischaemia-induced neovascularisation in eNOS-deficient mice bestows a key role on NO in mobilising EPCs. By inducing the phosphorylation of eNOS, VEGF plays an important role in stimulating NO production, a relationship confirmed by increases in the peripheral EPC count in normal mice after VEGF administration but not in eNOS-deficient mice [105]. This study also shows that exogenous EPC delivery improves limb function and recovery in “healthy” mice in that the NO-mediated activation of MMP-9 appears to be critical [106]. Activated MMP-9 promotes the transformation of insoluble membrane-bound Kit ligand into its soluble arrangement (sKitL), allowing for the movement and mobilisation of EPCs from the BM into circulation [96].

Another pathway linked to the neovascular effects of EPCs is Notch1, a transmembrane receptor. Notch1 and its ligand Jagged1 have been implicated in post-ischaemic neovascularisation in both experimental and clinical stroke, where increases in the expression of activated Notch1 (Notch intracellular domain or NICD) in peri-infarct endothelial cells are coupled with the level of angiogenesis [107]. Neo-angiogenesis occurs by the proliferative sprouting of endothelial tip cells, followed and stabilised by endothelial stalk cells. Notch1 signalling co-ordinates this motility between tip and stalk cells and possibly directs arterial EC differentiation [108]. This relationship is supported by the suppression of tumour growth via the inhibition of Notch signalling [109]. NICD may also regulate transcription and allow physical cellular changes to take place during angiogenesis [108]. Defects in EPC mobilisation contribute to neovascularisation impairment in ischaemic tissue, again supporting the association between EPCs and their role in vascular remodelling [106].

Whilst there are many pre-clinical studies scrutinising the safety and efficacy of EPC treatment in stroke, only a few clinical studies have investigated the therapeutic efficacy of autologous EPCs in stroke patients. For instance, a recent phase I/IIa study examining the effects of autologous EPC transplantation in eighteen acute ischaemic stroke patients has reported this approach to be safe with no sign of any increased tumourigenicity or any other adverse events over the four-year follow-up period. Though no improvements in neurological outcomes were measured, the lower incidence of adverse reactions suggests a certain degree of efficacy in improving the quality of life. The pre-conditioning of treatment may be considered here, where the upregulation of CXCR4 could become a therapeutic target in enhancing EPC efficacy [110]. Other attempts to increase HIF-1 and VEGF also concluded that the overexpression of such genes augmented neovascularisation, proving therapeutic benefit as a result of EPC priming [28,97]. 

## 7. Haematopoietic Stem Cells 

HSCs are multipotent, tissue-specific stem cells able to give rise to all functional blood cell types, including leukocytes, erythrocytes, and thrombocytes. HSCs present treatment possibilities as their supplementation encourages the recovery of diseased tissue by restoring blood and oxygen flow. The regeneration of ischaemic cells is facilitated by HSC differentiation (haematopoiesis), a process regulated by several hormones and cytokines, namely EPO, IL-3, granulocyte colony-stimulating factor (G-CSF), and macrophage colony-stimulating factor (M-CSF) [111]. CD34, though a surface marker expressed by other cells, is generally understood to represent hematopoietic stem and hematopoietic progenitor cells [112]. CD45 is another notable marker of HSCs [113]. HSCs can be obtained from the BM, peripheral blood, and umbilical cord blood. Where engrafting HSCs from BM-MNCs allows for faster expansion, they express higher human leukocyte antigen (HLA) levels, creating problems with rejection during allogeneic therapy. Conversely, cord cells are more immature and flexible in HLA matching but are lower in concentration [114]. 

The human adult produces over two hundred billion red blood cells per day [115]. With such a high turnover rate, the proliferative abilities of stem cells are most vitally exercised here where cell fate, regarding self-renewal or differentiation, is determined by gene expression and regulated by transcriptional factors [116,117]. Regulators between the two pathways are not distinct or separate, with factors able to influence cell fate down either route. However, some lineage-specific growth factors, such as G-CSF, M-CSF, and EPO, are categorical in directing HSCs down their respective pathways [118]. At high concentrations, GATA-1 suppresses the HSC exosome complex, consequently arresting early erythroblast proliferation and thus allowing for their maturation [119]. Conversely, GATA-1 downregulation is an important molecular cue in terminal erythroid maturation [120].

The Wnt and Notch pathways are other regulators of haematopoietic cell fate. Both Wnt and Notch receptors are widely expressed throughout the haematopoietic system and are critical in co-ordinating the development of leukocytes and their divisions. Wnt3a and Notch signalling promote early T-cell differentiation in human umbilical cord (hUCB) blood stem cells. Conversely, the inhibition of Wnt in the presence of Notch instead directs HSCs to give rise to natural killer cells [121]. It is necessary to understand the signalling that occurs during HSC differentiation as it allows better identification of potential targets to enhance the therapeutic efficacy of stem cell application in future trials.

Aside from transcriptional signalling, external situations also drive haematopoietic cell fate. For example, erythropoiesis occurs when HIF is activated under oxidative stress [122]. A study with MCAO rats provided insight into this relationship, where rats which intracerebrally received a culture of hypoxia-exposed (3% O_2_) HSCs displayed significantly better neurological outcomes compared to those which received normoxia-exposed (20% O_2_) or no treatment at all. This study also showed the role of exchange protein Epac1 in regulating the HIF/MMP pathway, with evidence connecting this communication to the promotion of neural progenitor cell (NPC) homing, aiding cerebral neuroplasticity. These results confirm previous findings documenting Epac1 action to enhance MMP activity and promote neovascularisation through the integrin-mediated adhesion of circulating HSCs to endothelial layers [123]. CD45+ bone marrow mononuclear cells (BMMNCs) were shown to differentiate into endothelial cells and smooth muscle cells to promote angiogenesis in an ischaemic stroke rat model [124]. Other pre-clinical studies have also reported enhanced angiogenesis as a result of HSC treatment [125,126].

Analysis of an “open-labelled nonrandomised phase I/II trial” investigating the safety of G-CSF treatment in patients with acute ischaemic stroke allowed for broader observations of HSC treatment. Subcutaneous G-CSF administration was deemed safe and feasible; however, the small cohort could not infer a clear dose-response relationship between G-CSF and related HSC mobilisation. The improvements in neurocognitive performance, long-term memory, and attention observed in this study may derive from the adjunctive contribution of IV thrombolysis present in this study [127]. 

The first clinical trial measuring IA autologous CD34+ stem cell delivery in human ischaemic stroke patients found that intervention was well tolerated by all participants (primary outcome measure), with improvements in clinical function characterised by a significant decrease in mean NIHSS score from 10.40 to 2.20 (95% CI; 3.69–12.71; *p* = 0.007) [128]. In addition, reductions in lesion volume were also observed, furthering evidence of neuroprotection. A separate trial into the intrathecal delivery of CD34+ stem cells also deemed this safe and did not lead to any allergic or immunological AEs [129]. Improvements in neurological scores, as assessed by NIHSS and BI, were again observed. These promising results and those of other studies [130,131,132,133] prompted STROKE34 (EU clinical trial registration: 2017-002456-88), a randomised controlled phase IIa trial of IA CD34+ cells in acute ischaemic stroke [134]. Its primary outcome looks to measure infarct volume with magnetic resonance imaging (mRI) at three months, with secondary outcomes looking into the safety and efficacy of the treatment. The results of the trial are expected to shed some light on the direction of future studies with HSCs in the field of stroke medicine.

Finally, in an attempt to augment therapeutic benefit, one study explored the idea of the genetic editing of HLA expression in HSCs [135]. The downregulation of HLA-A lifts restrictions on current HSC-based therapies as the risk of rejection for allogenic transplantation is reduced. This increases the suitability of treatment in cases where the patient’s HLA profile is under-represented in the current donor pool. 

## 8. Neural Stem Cells 

NSCs are undifferentiated stem cells of the CNS. They are multipotent stem cells able to self-renew and proliferate, give rise to different cell types, and differentiate into the three cell types of neural lineage, neurones, astrocytes, and oligodendrocytes [136]. Neurones, simply, are electrically excitable cells that synaptically transmit signals throughout the body [137]. Glial cells support and define these communications and are categorised by their functions; astrocytes maintain an appropriate chemical environment for brain functionality, and oligodendrocytes are responsible for myelination [138]. NSCs are sometimes referred to in the literature as “NPCs”, “neural precursor cells”, or “radial glia”, terminology which is used interchangeably and tends to be a difference in semantics. For clarity, this review distinguishes NSCs from their progenitors by the differences in their capacity to proliferate and differentiate. Where NSCs can infinitely divide, NPCs are slightly more specialised with a limited number of replication cycles. NPCs also cannot give rise to non-neural cells present in the CNS, such as immune cells, whereas NSCs can [136].

NSCs originate from the neuroectodermal tissue of the neural plate and are primarily found in the ventricular–subventricular zone (V-SVZ) of the walls of the lateral ventricles and the subgranular zone (SGZ) of the dentate nucleus [139,140]. NSCs are isolated by the enzymatic digestion of these locations [141] and quantified either in vitro using Reynolds and Weiss’ method of Neurosphere assay or by using a more recently developed collagen-based assay, Neural Colony-Forming Cell (NCFC) assay [142]. NCFC assays are now more commonly used as they are efficient in multiplying NSC count and can also discriminate between NSC and NPC populations by analysing the sizes of the colonies, representative of their proliferative abilities, the assay produces [143].

Neurogenesis is the growth and development of neuronal tissue and occurs both prenatally and in adults. It is the process by which NSCs develop into either neurones or glial cells (gliogenesis) and is influenced by both internal and external factors. Extrinsic factors in the local microenvironment of the SVZ and SGZ determine the lineage of NSCs, with soluble factors and transcriptional factors controlling intracellular signalling cascades such as the Notch-Hes1 pathway [144,145,146]. The activation of such pathways, triggered by oxidative pressures, decides whether NSCs will transform into astrocytes and oligodendrocytes or differentiate into neurones. As discussed before, MSCs can also induce neurogenesis. bFGF and EGF initiate the self-differentiation of MSCs into NPCs [46] or direct the differentiation of already established NSCs into astrocytes or neurones [44].

By replacing necrotic neurones and positively influencing neuroregenerative pathways adversely affected by ischaemia, NSCs, through neurogenesis, present an exciting therapeutic option. The migration and differentiation of NSCs into mature neurons have been shown to restore cerebral homeostasis in MCAO rats [147]. Other therapeutic actions of NSCs, such as those including the modulation of the immunomodulatory response, reorganisation of neuronal pathways, and angiogenesis, somewhat resemble that of MSCs. The immunomodulatory properties of NSCs are supported by a marked attenuation in BBB damage, reduced cytokine production, and expression of proinflammatory markers IL-6 and TNF-α observed in acute stroke mice injected with a mixture of human-induced pluripotent stem cells (iPSCs) and NSCs in the hippocampus [148]. The behavioural improvements observed in these mice were comparable to those noted by other studies [149,150]. In another study, improvements in the behavioural scores of NSC-treated mice appeared to correlate with the extent of angiogenesis and reduction in infarct volumes [151]. The neuroprotective effects of NSCs are further recognised in a study of pre-conditioned cells in magnetically targeted MCAO rats [152]. The study found that, compared to the other treatment groups, the pre-conditioned NSCs demonstrated better migration and differentiation capacity as well as comparable improvements in neurological function. Taken together, these studies reveal a range of NSC-related benefits ranging from cell replacement to enhanced vascularisation, thereby proving the ability of NSCs to induce improvements in functional outcomes and neuronal reorganisation.

Similar to translational studies, treatments with NSCs have led to improved mental status, limb strengthening, and speech recovery in clinical settings. These were inevitably associated with marked improvements in overall quality of life compared to the control group and functional benefits further manifested in improvements in functional status [153].

The Pilot Investigation of Stem Cells in Stroke (PISCES) trials are a collection of clinical studies looking at NSC treatment for ischaemic stroke. In response to a successful pre-clinical trial in which CTX-DP (a manufactured product as a suspension composed of CTX0E03 cells at a concentration of 5 × 10^4^ cells/μL) yielded sensorimotor improvements in MCAO rats, an outsetting phase-I, open-label, dose-escalation study into the safety and tolerability of CTX-DP was conducted in human stroke patients [154]. The trial was thorough in its endeavours, analysing eleven men at a range of doses (three patients receiving two million CTX0E03 NSCs; three other patients receiving five million; three others receiving ten million; two others receiving twenty million) at a mean time of twenty-nine months (range from 6 to 60 months) after stroke onset. The primary endpoint was safety, measuring the emergence of any serious AEs to which no treatment-related adversities were found, thus proving safety up to twenty million CTX0E03 NSCs. Though changes in NIHSS and mRS scores suggested some degree of neurological and functional improvement, the trial concluded that the small patient population and nature of its design limited the reliability of these conclusions, precluding further investigations.

PISCES-2 also reflected this requisite for additional research [155]. The intracerebral implantation of CTX0E03 NSCs (dose twenty million) was deemed feasible and safe, and improvements in the Action Research Arm Test (ARAT, an evaluative measure to assess limb function among individuals with cortical damage) were seen in a total of four patients (17%) by twelve months after implantation. It was noted that these improvements were only found by those who initially demonstrated residual upper limb control and not by anyone with absent upper limb movement at baseline. PISCES-3 (trial registration: NCT03629275) began in August 2018 and was the natural continuation of PISCES-2. Unfortunately, the PISCES-3 trial was terminated due to the COVID-19 pandemic. Whilst a few other clinical trials are assessing NSCs, there is evidence in other areas to prove that the transplantation of neurones themselves is safe and feasible [156,157]. However, no clinical benefit was found in these cases, perhaps due to the therapeutic limitations of mature neuronal cells. 

**Table 1:** Translational and clinical studies employing stem cells as therapeutics.

Cell Type	Dose	Route of Administration	Timing of Treatment (Post-Model Onset)	Participants	Outcome Assessment	References
Pre-clinical research						
AD-MSCs	2 × 10^6^	IV	2–7 d	44 MCAO rats	Safe; improved sensorimotor function	[56]
BM-MSCs	2 × 10^6^	IV and IP	3 h–7 d	36 MCAO rats	Significantly improved neurological function	[59]
BM-MNCs	3 × 10^6^	IV	4 d	71 rats	Improved cognitive function	[55]
MSCs	1 × 10^5^	IA	6 h	MCAO rats	Improved functional outcome	[60]
MSCs and EPO	2 × 10^6^	IV	24 h	Focal ischaemic rats	Increased neurogenesis	[61]
EPCs	4 × 10^6^	IV	ND	Hind limb ischaemic rats	Improved limb function	[106]
EPCs	4 × 10^6^	IV	24 h	MCAO rats	Improved functional outcome	[158]
ECFCs and EPO	5 × 10^6^	IV and IP	24–72 h	Focal ischaemic rats	Improved neurological function	[159]
ECFCs	1 × 10^6^	IA	72 h	Focal ischaemic mice	Improved neurological function	[160]
hUCB-HSCs	1 × 10^6^	Intracerebral	7 d	MCAO rats	Improved neurological function	[123]
iPSC-NSCs	1 × 10^6^	Intra-striatal	7–14 d	15 MCAO mice	Improved behavioural and sensorimotor function	[147]
iPSC-NSCs	1 × 10^5^	Intra-hippocampal	24 h	MCAO mice	Improved neurological function	[148]
NSCs	1 × 10^5^	Intracerebral	24 h	MCAO mice	Behavioural improvement	[149]
NSCs	3 × 10^6^	IV	6 h	15 MCAO rats	Neuroprotective effects	[150]
NSCs	1.2 × 10^5^	Intracerebral	24 h	7 MCAO rats	Increased vascularisation	[151]
NSCs	4 × 10^6^	IV	24 h	23 MCAO rats	Improved neurological function	[152]
HSCs	5 × 10^5^	IV	48 h	Transient ischaemic mice	Increased neovascularisation	[125]
OECs	4 × 10^6^	IV	24 h	MCAO mice	Decreased brain oedema volume	[77]
Clinical trials						
Acute phase						
BM-MNCs	4–6 × 10^8^	IV	24–72 h	10	Safe; clinical improvements	[131]
HSCs	2.5–10 µg/kg	Subcutaneous injection	12 h	20	Safe; neuropsychological improvements	[127]
Subacute phase						
HSCs	5.1 × 10^7^–6 × 10^8^	IA	3–7 d	20	Safe; clinical improvements	[161]
HSCs	1.59 × 10^8^	IA	5–9 d	10	Feasible and safe	[132]
HSCs	1 × 10^8^	IA	7 d	5	Safe; significant clinical improvements	[128]
BM-ALDH^br^ stem cells	1.6 × 10^5^–7.5 × 10^7^	IA	11–19 d	29	Safe	[31]
UC stem cells	5 × 10^6^–5 × 10^7^/kg	IV	3–10 d	10	Safe and feasible	[162]
HSCs	6.1 × 10^8^	IA	8–15 d	10	Safe; good clinical outcome	[69]
HSCs	2.8 × 10^8^	IV	18 d	58	Safe	[133]
HSCs	3 × 10^7^	IA	9 d	1	Feasible	[130]
MSCs	1 × 10^8^	IV	ND	5	Safe	[163]
Chronic phase						
BM-MSCs	1 × 10^8^ (*n* = 10) 3 × 10^8^ (*n* = 10)	IV	1 m	16	Safe; behavioural and physiological improvements	[65]
MSCs and NSCs	0.5–6 × 10^6^/kg	IV and intracistern	<1 wk–2 yrs	6	Safe	[71]
MSCs	0.5–1.5 × 10^6^/kg	IV	>6 m	36	Safe; behavioural improvements	[164]
EPCs	5 × 10^6^/kg	IV	4–6 wks	18	Improved long-term safety	[165]
NSCs (CTX-DP)	0.2–2 × 10^7^	Ipsilateral putamen injection	6–60 m	11	Safe; improved neurological function	[154]
NSCs	2 × 10^7^	Intracerebral	2–13 m	23	Improvements in upper limb function	[155]
BM-MSCs	2.5 × 10^6^–1 × 10^7^	Intracerebral	6–60 m	18	Safe; significant clinical improvements	[68]
HSCs	0.8–3.3 × 10^7^	Intrathecal	1–7 yrs	8	Safe; improved clinical neurological function	[129]
Neurones	0.5–1 × 10^7^	ND	6 m–6 yrs	26	Safe and feasible	[156,157]
US-MSCs	2 × 10^7^	IA	<3 m	3	Safe; improved neurological function	[166]
NSCs and HSCs	2 × 10^8^	Intracerebral	ND	10	Safe; functional improvements	[153]
BM-MSCs/HSCs	5–6 × 10^7^	IV	3 m–2 yrs	20	Safe; significant functional improvements	[64]
NPCs and UC-MSCs	0.2–2.3 × 10^7^	Intraparenchymal	6 m–20 yrs	10	Safe; functional improvements	[70]
MSCs	0.2–2.3 × 10^8^	IV	<6 m	12	Safe and feasible	[66]

AD, adipose tissue; MSC, mesenchymal stem cell; MCAO, middle cerebral artery occlusion; BM, bone marrow; EPO, erythropoietin; ECFC, endothelial colony-forming cell; hUCB, human umbilical cord blood; iPSC, induced pluripotent stem cell; MNCs, mononuclear cells; NPCs, neural progenitor cells; ND, no data.

## 9. Route, Dose, and Timing of Treatment

### 9.1. Route

The main routes for treatment are IV, IA, and intracerebral administration. Both the pre-clinical and clinical studies show the IV route as the most preferred route due largely to its ease of use and its non-invasive nature. However, the IV treatment poses issues with engraftment and therapeutic efficacy due to the clearance of most cells by the lungs and liver during circulation [167,168]. Furthermore, chemotaxis signalling subsides over time which suggests that IV administration may be more suitable in treating acute and subacute stroke where the levels of inflammatory biomarkers are at their highest, compared to chronic cases. Even so, studies demonstrating the efficacy of IV administration in chronic settings also exist, supporting the fundamental property of stem cells in their ability to home to sites of injury [164]. Additional references for this can be found in Table 1.

IA administration is similar in technique as a minimally invasive and straightforward procedure. It is argued the IA route is more efficient than IV transport as this route does not lead to excessive cell trapping. While some studies comparing routes of stem cell delivery favour the IA route over IV administration [169], others report that there is no real difference in efficiency, with both routes revealing similar biodistribution rates and comparable functional outcomes [170,171,172]. 

Another route mentioned is the intracerebral route. The direct administration to site of injury eliminates the need to rely on chemical paracrine signalling in directing stem cell migration, allowing for smaller dose deliveries. This also, in theory, makes it a better option for chronic stroke patients (where the homing of stem cells may be weaker due to the absence of inflammatory mediators attracting as such) to maximise stem cell transfer; however, not all targets are physically accessible. Its neuronal nature has been associated with therapeutic benefits; however, its intrusiveness increases the risk of adversities, including infection and haemorrhage [173].

**Figure 2:**
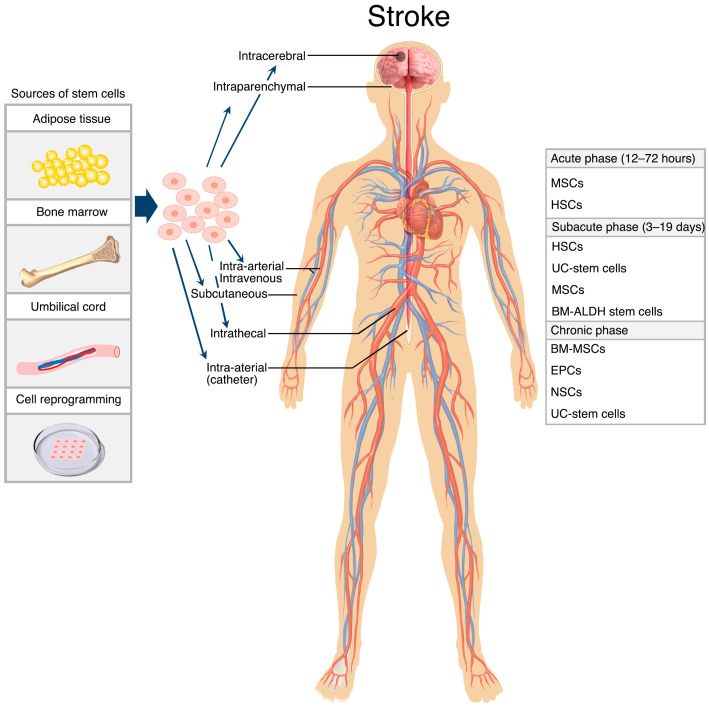
Different sources, routes, and time windows for therapeutic stem cells. The figure summarises the most commonly used stem cells obtained from adipose tissue, bone marrow, umbilical cord, and laboratory reprogramming in clinical studies. Despite the availability of different routes for administration, intravenous and intra-arterial routes remain the most preferred routes, particularly during the acute phase of stroke.

Other routes include intrathecal and intraperitoneal administration. However, little is known about the efficacy and overall suitability of these routes due to the limited availability of studies employing them. When organising management strategies for ischaemic stroke patients, concerns of safety, the stage of stroke, and other practical measures must be considered. Although there is little evidence comparing the effectiveness of routes at different stages, it is reasonable to think that IV and IA routes may prove greater therapeutic effect in the acute and subacute phases of the disease [69,128], whereas IC administration may be better suited for patients in the chronic phase of the disease [68,154,155]. Stem cell sources, administration routes and time windows of stem cells therapy have been summarised in Figure 2. 

### 9.2. Dose

Despite the investigation of a wide range of cell concentrations in various clinical and pre-clinical studies, the optimal dose for an effective therapy after a cerebral ischaemic event continues to be a matter of debate. The lack of AEs at all doses tested negates the concerns regarding the numbers of stem cells to be administered and suggests the consideration of the reported efficacy of cells at a particular dose for a particular stem cell type. Though no clinical studies specifically evaluate the differences in stem cell efficacy at different concentrations, several studies comment on the safety over a range of cell doses. In the studies discussed in this paper, the doses of cells administered varied from 0.5 × 10^5^ cells/kg to 6.1 × 10^8^ cells [69,164]. In clinical trials, higher cell doses appear generally to be associated with better outcomes and lower transplant-related mortalities. However, as alluded to above, it is impossible to draw a conclusion about the relationship between treatment concentrations and efficacy [65,68,69,128]. Functional and neurological improvements in pre-clinical trials were observed throughout the range of 1 × 10^5^–5 × 10^6^ cells/kg, suggesting that an optimal dose may lie within [60,126,149,159]. 

The optimal dose of stem cell treatments is likely to be dependent on the cell types and administration routes. It is important to remember that certain routes, notably IV injection, will lead to major losses in stem cell numbers due to their trapping by the lungs [174]. Therefore, a higher dose and repeated injection may be necessary while using these routes [175]. 

### 9.3. Timing

One of the biggest arguments for investing time and resources into stem cell research is the hope that the emerging treatment option(s) will demonstrate a larger therapeutic window than the current time limitations. The short life span of rodents is an issue when considering long-term intervention, explaining why pre-clinical studies fail to produce data on optimal treatment timing. Clinical trials, however, can evaluate the safety, feasibility, and efficacy of treatments with stem cells over a significant period of time. In addition, the time of administration varies significantly in clinical studies, ranging from twelve hours to twenty years, where safety is confirmed throughout [70,127]. 

There is little clinical evidence as to the application of stem cells during the hyperacute phase of stroke, so it is difficult to establish a consensus on the optimal timing of treatment in the immediate aftermath of stroke. In contrast, several clinical studies with acute stroke patients exist. They unanimously show that patients who received stem cells 7–72 h after stroke onset displayed better neurological outcomes [127,131]. A multicentre phase 2 clinical trial showed improved outcomes in acute ischaemic stroke patients who received intravenous multipotent adult progenitor cells within the first 36 h of stroke, suggesting greater benefits of early interventions with stem cells [176]. Clinical improvements with stem cell therapy are also commonly observed during the chronic phase of stroke, even up to 5 years after stroke onset [68,154,155,164]. Pre-clinical studies, which solely investigate the efficacy of stem cell treatment in hyperacute or acute stroke models, replicate these findings, report improved outcomes, and strengthen the argument that earlier treatments provide greater benefits. Even so, the current evidence does not strongly associate the degree of therapeutic efficacy with the timing of treatment and implies a need for future studies. 

### 9.4. Comparison of Treatments with Different Types of Stem Cells

It is likely that treatments with different types of stem cells may yield different effects in the same disease settings which, in some cases, may be complementary. At present, not many clinical studies, if any, comparatively assess the therapeutic impact and safety of different stem cells in the same patient group. Future studies specifically exploring this issue in ischaemic stroke patients are likely to provide invaluable information as to the efficacy of different stem cells. They may also provide additional information about the dose and timing of administration of different stem cells. The clinical features of treatments with different stem cells are summarised in Table 2.

## 10. Discussion and Conclusions

This paper set out to discuss the potential role of different types of stem cells as therapeutics for ischaemic stroke. Pre-clinical and clinical data analysed throughout the text provide insight into the current position of treatments with stem cells in ischaemic stroke. The evaluation of regenerative processes such as angiogenesis, neovascularisation, neurogenesis, and erythropoiesis explains the ways in which stem cells act through differentiation and proliferation to recover ischaemic tissue. In addition, analysis of the (paracrine) signalling that directs these activities further helps to guide our understanding of stem cells as therapeutics. 

Stem cell therapy has been widely used in various diseases such as leukaemia, myeloma, neurological degeneration, or vascular diseases. In addition to minor side effects such as nausea, headache, or low-grade fever, few severe adverse events (SAEs) have also been reported in patients who received stem cells as therapeutics. These include infection, allergic reaction, arrhythmia, thromboembolism, fibrosis, immune rejection, and transplantation-surgery-related intracranial haemorrhage [177,178,179]. Even fewer cases of SAEs, e.g., seizure, arrhythmia, and intracranial haemorrhage, have been reported in ischaemic stroke patients treated with stem cells as compared to matching placebo groups [65,71]. Indeed, safety and feasibility are conclusively measured in all the relevant studies cited in this review, and it is evident that intervention with stem cells for ischaemic stroke does not induce any major AEs. Severe ischemic stroke can also result in the above-mentioned SAEs. Clinical studies with larger sample sizes are necessary to further ascertain the safety of treatments with different stem cells. 

In terms of the therapeutic efficacy of stem cell application, the majority of the pre-clinical studies yielded positive results ranging from enhanced angiogenesis to improved sensorimotor functions. Similarly, a number of clinical studies have also reported clinical improvements in long-term follow-up from 6 months to 2 years [65,69,127], while others failed to document any neurological or functional benefit [31,71,132,165]. This may in part be due to the fact that in pre-clinical studies acute interventions aiming to rescue ischaemic tissue present fewer challenges (with chemotaxic signalling and viability of penumbra at their highest) than late interventions in chronic stroke in clinical settings. It could also be due to the design limitations of early-phase trials, where research is restricted in its endeavours (Phases of Clinical Trials, 2019). Phase III and later trials may properly abet investigations into treatment efficacy with more time, resources, and larger patient cohorts. Trials like “Umbilical cord-derived Mesenchymal Stem Cells for Ischaemic Stroke (UMSIS; NCT04811651): a Prospective, Double-blinded, Randomized Controlled, Pilot Study” may provide insight into the direction of future clinical trials. With an estimated enrolment of two hundred participants, UMSIS is one of the largest trials in the area. It is a quadruple-masked, randomised parallel assignment monitoring the effects of IV-injected umbilical cord MSCs (1 × 10^8^ cells). The trial evaluates functional improvements by primarily comparing mRS scores before and after treatment. It will also examine other clinical measures such as changes in the FM scale and NIHSS score. 

Methods and ideas surrounding treatment priming have also gained attention in regenerative stroke research. Another option for future practice may involve mixed cell approaches, whereby conjunctive therapy using multiple stem cell types promises to target a range of pathologies [180,181]. Research may also benefit from investigations into the parameters of route, dose, and timing of administration to create a standard strategy for stem cell treatment, providing an interventional framework available for case-to-case manipulation. 

Due to a gap in the literature, it is difficult to reflect on the other elements of treatment, such as cost-effectiveness and the practicalities of cell proliferation. Ethics is another important consideration of stem cell research. Though the use of adult and cord blood stem cells are less topical than embryonic sources, they still raise regulatory concerns over genetic manipulation [182]. Albeit somewhat crucial for successful allogeneic therapy [164], genetic editing may not completely eradicate the risk of rejection.

Pre-clinical or translational studies constitute important prerequisites for proving or disproving the therapeutic action or capacity of any given agent. They provide preliminary data on the desired biological effect (efficacy) and associated toxicities (safety) of drugs which ultimately inform the design of subsequent clinical research [183]. The majority of pre-clinical studies investigating the impact of stem cell therapy for stroke utilise a rodent model of human ischaemic stroke, achieved by the temporary or permanent occlusion of the middle cerebral artery (MCAO) [184,185,186]. Damage occurring in the cortex and subcortical structures like the thalamus and striatum in this model reflect the pathology observed in clinical settings. Several clinical trials with stem cells in ischaemic stroke have shown the safety and effectiveness of this approach in humans [65,69,162,165]. However, for the progression of clinical studies into later-phase trials (phase III onwards), treatment safety must be confirmed.

In conclusion, stem cell treatment presents possibilities for patients with all types of ischaemic stroke. With evidence of safety and efficacy measured in patients with acute, subacute, and chronic disease, therapeutic interventions appear to be promising for patients at every stage of the disease (Table 1). However, further clinical research is necessary to standardise the treatment regimens.

## Figures and Tables

**Table 2:** Characteristics of different stem cells in stroke treatment.

Stem Cell Type	Dose	Route	Timing	Adverse Effects	Outcomes	References
MSCs	2 × 10^6^–3 × 10^8^	IA IV Intracerebral	1 week–60 months	Headache Nausea Seizure	Safe Improved clinical outcome Improved motor recovery	[65,68,71,163,166]
HSCs	3 × 10^7^–6.1 × 10^8^	IA IV Intrathecal Intracranial	12 h–7 years	Fever Hepatic damage Seizure	Safe Improved clinical outcomes	[69,127,129,130,133]
EPCs	5 × 10^6^/kg	IV	4–6 weeks	Seizure Deep vein thrombosis Arrhythmia	Safe	[165]
NSCs	2 × 10^6^–2 × 10^8^	IV Intracranial Intracerebral	1–60 weeks	Low fever Infection Headache	Safe Improved neurological function	[71,153,154,155]
